# Causal Factor Disentanglement for Few-Shot Domain Adaptation in Video Prediction

**DOI:** 10.3390/e25111554

**Published:** 2023-11-17

**Authors:** Nathan Cornille, Katrien Laenen, Jingyuan Sun, Marie-Francine Moens

**Affiliations:** Language Intelligence and Information Retrieval (LIIR) Lab, Department of Computer Science KU Leuven, 3001 Leuven, Belgium; katrien.laenen@kuleuven.be (K.L.); jingyuan.sun@kuleuven.be (J.S.); sien.moens@kuleuven.be (M.-F.M.)

**Keywords:** causal representation learning, video prediction, transfer learning, few-shot learning

## Abstract

An important challenge in machine learning is performing with accuracy when few training samples are available from the target distribution. If a large number of training samples from a related distribution are available, transfer learning can be used to improve the performance. This paper investigates how to do transfer learning more effectively if the source and target distributions are related through a Sparse Mechanism Shift for the application of next-frame prediction. We create Sparse Mechanism Shift-TempoRal Intervened Sequences (SMS-TRIS), a benchmark to evaluate transfer learning for next-frame prediction derived from the TRIS datasets. We then propose to exploit the Sparse Mechanism Shift property of the distribution shift by disentangling the model parameters with regard to the true causal mechanisms underlying the data. We use the Causal Identifiability from TempoRal Intervened Sequences (CITRIS) model to achieve this disentanglement via causal representation learning. We show that encouraging disentanglement with the CITRIS extensions can improve performance, but their effectiveness varies depending on the dataset and backbone used. We find that it is effective only when encouraging disentanglement actually succeeds in increasing disentanglement. We also show that an alternative method designed for domain adaptation does not help, indicating the challenging nature of the SMS-TRIS benchmark.

## 1. Introduction

Applications such as self-driving cars [1], weather prediction [2], or camera-equipped household robots [3] all benefit from the accurate prediction of high-dimensional future timesteps such as video frames or satellite imagery. A particularly challenging task is to make such predictions accurate when there are few samples to learn from. While it is typically expensive to gather enough frames from a new environment, many frames of a different-but-related environment *are* often available (such as a different location or different sensory equipment). This motivates us to propose a method to better enable a learner to make use of the related ‘old’ (in-domain or ID) data to increase prediction accuracy on the target ‘new’ (out-of-domain or OOD) dataset.

A naïve approach is simply training a model to be as accurate as possible on the ID data and subsequently fine-tuning it on the OOD data. Researchers have tried to improve on this approach by making certain assumptions about *how* the ID and OOD data are related. If *y* is the prediction target and *x* the input, typical assumptions are that only P(x) changes but P(y|x) stays the same (‘covariate shift’), or that P(y|x) changes but we can split *x* into $x_{variant},x_{invariant}$ such that $P(y|x_{invariant})$ stays the same [4,5,6,7]. One type of relatedness between the ID and OOD data is still underexplored, however, except in the case where there is a Sparse Mechanism Shift [8]. In this setting, the high-dimensional observables (like video frames) are views of lower-dimensional factors (such as the position of a pedestrian) evolving over time according to causal mechanisms that are fixed within one distribution (e.g., whenever the traffic light turns green, the pedestrians in front of it move forward in subsequent timesteps). The ID and OOD distributions, then, are different in only a small subset of these mechanisms. It is a technique that is able to improve the speed of the adaptation under the Sparse Mechanism Assumption, which can have significant implications for real-world applications. For example, when a robot undergoes a change in a sensor or actuator [9], such a technique would improve how quickly it can accurately predict future observations again. It can also help in a biological context by improving the predictability of gene expressions in new pathways, where the Sparse Mechanism Shift assumption has shown to be useful for disentangling biological processes [10]. The reasonableness of the Sparse Mechanism Shift hypothesis is motivated in [8]. In this work, we will take this hypothesis as true and explore how to improve few-shot prediction accuracy in a scenario that is true.

We develop a novel benchmark to test few-shot domain adaptation when the Sparse Mechanism Shift is true, called “SMS-TRIS”. It is derived from the TRIS datasets [11], which are synthetic datasets of video frames rendered from latent causal factors that evolve over time via stationary causal mechanisms, along with occasional interventions on the factors. SMS-TRIS consists of variants of the TRIS datasets in which one stationary mechanism is different per variant. We exploit the fact that the ID and OOD data are related through a Sparse Mechanism Shift by disentangling certain model parameters with regard to the true causal mechanisms. Specifically, we use CITRIS [11] to achieve this disentanglement, a method that leverages access to intervention information to guarantee disentanglement. If this succeeds, then a model of the ID data only needs to update the parameters that correspond to the subset of mechanisms that shifted. Based on the results from [12], this is hypothesized to increase speed-of-adaption, as a smaller number of parameters need to be updated, reducing the effective dimensionality of the hypothesis search space.

Experimental results indicate that the CITRIS extensions can improve performance, but that this improvement is brittle: it is dependent on the particular dataset, as well on the particular backbone (i.e., the basic model architecture to which the CITRIS extensions are added) that is used. Specifically, we find that the CITRIS extensions only improve the prediction performance when they succeed in significantly improving disentanglement.

A comparison with an alternative Domain Adaptation method (Deep CORAL [13]) indicates that it is not consistently effective either, indicating that SMS-TRIS is a challenging benchmark.

In summary, our contributions are as follows:

- We generate and make available SMS-TRIS, a new benchmark for evaluating few-shot domain adaptation for next-frame prediction under the Sparse Mechanism Shift assumption (The code to generate the datasets and reproduce our experiments as well as links to the dataset artifacts can be found at https://github.com/Natithan/SMS-TRIS (accessed on 21 August 2023.)
- We show that encouraging disentanglement during pretraining for few-shot domain adaptation can benefit prediction accuracy, but only if the disentanglement encouragement succeeds in leading to strong disentanglement.
- We show that an external baseline (Deep CORAL) does not improve over a naïve baseline, underlining the need for customized algorithms to exploit the Sparse Mechanism Shift assumption.

The remainder of the article is structured as follows: We position our work in the context of related work in Section 2. We detail our problem setup and explain our method in Section 3. We then describe the datasets and models we use in our experimental setup in Section 4. Finally, we discuss our experimental findings in Section 5 and conclude in Section 6.

## 2. Related Work

### 2.1. Causal/Disentangled Representation Learning

While the focus of our study is on leveraging disentanglement to improve few-shot performance, disentangling latent variables has often been of interest as a goal in itself. CITRIS is proposed by [11], with the goal of creating latent representations that are disentangled with regard to true causal factors. We build on this by evaluating the usefulness of such disentanglement for domain adaptation. Another work that disentangles latent representations using extra information is iVAE [14], which we include as a baseline under the name “Standard VAE”. Work such as [15] aims to improve disentanglement, stating that this can be useful for transfer learning or zero-shot learning. However, they assume independent factors of variation, which is not realistic when factors of interest can have common causes, as in our data. In [12], causal representation learning and domain adaptation are linked, but with reversed goals: they make use of speed-of-domain adaptation to achieve disentanglement with regard to the underlying causal structure; we achieve this disentanglement through other means (i.e., making use of intervention vectors as CITRIS does) and subsequently evaluate whether such disentanglement improves speed-of-domain adaptation.

### 2.2. Causality for Distribution Shifts

Researchers have investigated whether ideas from causality can benefit performance after a distribution shift. In [8], the authors motivate how causal representation learning could be relevant for machine learning purposes, such as domain adaptation, if the Sparse Parameter Shift assumption holds. However, they do not perform empirical experiments. There are a number of works [5,16,17,18] that make use of causal representation learning for distribution shifts, but they focus on domain *generalization* rather than domain adaptation. They typically have one mechanism of interest (that is, the target variable) and assume that this mechanism is invariant in distribution shifts, while the mechanisms of covariates are allowed to change. In contrast, this paper is interested in modeling mechanisms of multiple causal factors, assumes that a small number of them can change, and aims to improve the adaptation of the model to that change, rather than hoping to remain unaffected by it. Few-shot domain adaptation is performed in [4], but they assume known factors. They also do not assume a Sparse Mechanism Shift, but rather that no causal mechanisms shifted, and the distribution shift is due to a shift in the noise variable distributions. They then intend to learn a model that fits *both* the source-domain and target-domain data (a ‘conservative’ setting), while we intend to *adapt* the model (a ‘non-conservative’ setting). Moreover, they require access to multiple source domains.

### 2.3. Domain Adaptation

A substantial body of work shares the goal of performing domain adaptation, without using causality as a tool.

Many works focus on the classification setting, such as for images ([4,13,19,20] or for labeling activities in videos [21,22]). In Deep CORAL [13], the authors aim to improve the model performance on the OOD data based on access to the ID data and some OOD data. They align the covariance of latent features of the ID and OOD data, whereas we align the mechanisms and parameters for the ID data and investigate whether that improves the fine-tuning on the OOD data. It is complementary to encouraging disentanglement. We compared the effect of Deep CORAL to the effect of encouraging disentanglement in our experiments. A similar idea of aligning certain views of the samples from the source and target domain, but for videos, is used in [22]. Other works try to address domain adaptation for classification in a way that relies on having access to categorical labels [19,20,21]. Those techniques are not applicable for next-frame prediction, however.

Other settings in which domain adaptation has been tackled include semantic segmentation, language modeling, or keypoint detection [23]. In semantic segmentation, the meta-learning framework is common [24,25], in which training happens on a series of few-shot domain adaptation episodes, rather than the transfer learning setting in this work, in which we train on one big source domain and adapt to one target domain. In language modeling, parameter-efficient fine-tuning has been shown to be an effective approach for few-shot domain adaptation [26].

## 3. Methodology

We first detail the problem setup by formalizing the kind of data, the type of distribution shift, and the kind of models we consider. We then explain and motivate our method.

### 3.1. Problem Setup

The task under consideration is next-frame prediction, and for our purposes, we assume the frames come out of a Mechanisms-Induced Distribution:

**Definition 1** (Mechanisms-Induced Distribution)**.** *A Mechanisms-Induced Distribution D is characterized by the following:*

- *$P_{c_{0}}:R^{\sum_{k=1}^{K}\zeta_{k}}\to R^{+}$: A distribution from which the set of true, unobserved causal factors $c^{t}={\left(c_{k}^{t}\in R^{\zeta_{k}}\right)}_{k=1}^{K}$ at the first time t=0 are sampled, where $\zeta_{k}$ is the dimension of factor $c_{k}^{t}$;*
- *$P_{u_{X}}:R^{\zeta_{u_{X}}}\to R^{+}$: A distribution from which the rendering noise $u_{X}^{t}$ is sampled at each timestep, where $\zeta_{u_{X}}$ is the dimension of $u_{X}^{t}$;*
- *$P_{u}:R^{\sum_{k=1}^{K}\upsilon_{k}}\to R^{+}$: A distribution from which the set of factor noises $u^{t+1}={\left(u_{k}^{t+1}\in R^{\upsilon_{k}}\right)}_{k=1}^{K}$ is sampled at each timestep, where $\upsilon_{k}$ is the dimension of $u_{k}^{t+1}$. The $u_{k}^{t+1}$ are assumed to be independent of each other: $P_{u}\left(u_{1}^{t+1},\ldots ,u_{K}^{t+1}\right)=\prod_{k=1}^{K}P_{u_{k}}\left(u_{k}^{t+1}\right)$;*
- *$f_{k},k\in \left\{1\ldots K\right\}$: The true causal mechanisms that determine how factors evolve: $c_{k}^{t+1}=f_{k}\left(c^{t},u_{k}^{t+1}\right)$. They are stationary;*
- *$X^{t}$: The observed video frame at time t, deterministically ‘rendered’ from a combination of $c^{t}$ and $u_{X}^{t}$ by some observation function h: $X^{t}=h\left(c^{t},u_{X}^{t}\right)$.*

It is possible that a factor $c_{k}^{t+1}=f_{k}\left(c^{t},u_{k}^{t+1}\right)$ depends only on a subset of factors $Pa(c_{k}^{t+1})$:

(1) $$Pa\left(c_{k}^{t+1}\right)=\left\{c_{l}^{t}|1\leq l\leq K,\exists c^{t},u_{k}^{t+1}:\frac{\partial f_{k}\left(c^{t},u_{k}^{t+1}\right)}{\partial c_{l}^{t}}\neq 0\right\}$$

We call this subset the parents of $c_{k}^{t+1}$.

We assume the two distributions are related by a Sparse Mechanism Shift:

**Definition 2** (Sparse Mechanism Shift (SMS))**.** *We define a Sparse Mechanism Shift as a Boolean property of two Mechanisms-Induced Distributions, $D_{1}$ and $D_{2}$, and an integer s that is true if and only if $D_{1}$ and $D_{2}$ are equal in all characteristics except in the subset S of all K true causal mechanisms, $S=\{f_{i}|1\leq i\leq K,|S|=s\}$.*

In the rest of the paper, we will be informal and leave out the *s* argument when we just mean that *s* is a small fraction of all *K* mechanisms. We will model the data with Encoder-Transition models:

**Definition 3** (Encoder-Transition Model)**.** *An Encoder-Transition Model M is characterized by:*

- *An invertible encoder $e_{\theta }$, with inverse function $e_{\theta }^{-1}$, that maps between $X^{t}$ and a latent representation $z^{t}=e_{\theta }\left(X^{t}\right)$, ${\hat{X}}^{t+1}=e_{\theta }^{-1}\left({\hat{z}}^{t+1}\right)$.*
- *A transition prior $p_{\phi }$ that predicts the next timestep in the latent space: ${\hat{z}}^{t+1}=p_{\phi }\left(z^{t}\right)$.*

Such a two-component model is a natural fit when the true Mechanisms-Induced Distribution can also be partitioned along the observation function *h* and the mechanisms $f_{k}$.

### 3.2. Our Method: Causal Mechanism Disentanglement for Few-Shot Domain Adaptation

Our approach to improve few-shot next-frame prediction after a Sparse Mechanism Shift in order to encourage the parameters of the transition prior $p_{\phi }$ to being disentangled with regard to the true mechanisms $f_{k}$. We refer to such disentanglement as Causal Mechanism Disentanglement.

We will first detail Causal Mechanism Disentanglement and then discuss why it is expected to improve few-shot next-frame prediction after a Sparse Mechanism Shift.

#### 3.2.1. Causal Mechanism Disentanglement

**Figure 1:**
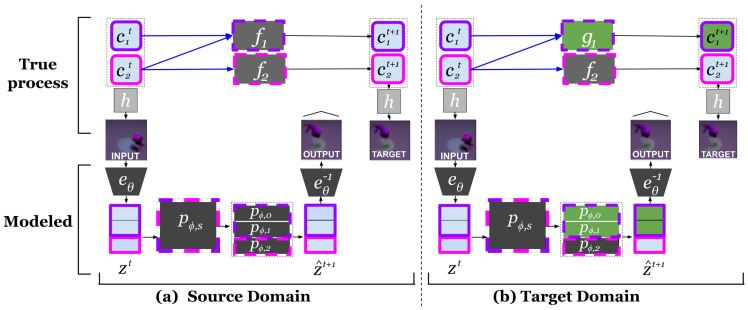
(**a**) Causal mechanism disentanglement for three latent dimensions of $z^{t}$ and two true causal factors $c_{k}^{t}$, with sample frames from the Shapes dataset. The top part shows the true factors and mechanisms along with the observation function *h* that produces the observed frames. The bottom part shows the modeled latents and parameters. The alignment between true factors $c_{k}^{t}$ and disentangled dimensions of the model activations $z_{i}^{t}$ is indicated with full bold-colored outlines (purple for factor 1 and $z^{t}$ dimensions 0 and 1; pink for factor 2 and $z^{t}$ dimension 2). (**b**) In the target domain, the shifted mechanism $g_{1}$, with a green background, leads to a shift in $P(c_{1}^{t+1}|Pa\left(c_{1}^{t+1}\right))$. If the encoder $e_{\theta }$ disentangles different causal factors into different subsets of the latent $z^{t}$, this leads to a shift only in $P(z_{\psi_{1}}^{t+1}|Pa\left(z_{\psi_{1}}^{t+1}\right))$. It is then possible to adapt to this change during transfer learning by updating only $p_{\phi ,\psi_{1}}$. This work evaluates whether this isolation of the parameters that need to update can be exploited to improve few-shot domain adaptation.

Figure 1a illustrates Causal Mechanism Disentanglement with regard to a Mechanism-Induced Distribution in an Encoder-Transition Model.

To define Causal Mechanism Disentanglement, consider the following variables:

- An Encoder-Transition Model *M* with encoder $e_{\theta }$ and transition prior $p_{\phi }$;
- A Mechanisms-Induced Distribution *D*;
- An assignment function ψ:J→K,J≥K that assigns dimensions of $z^{t}$ to an index *k* in {1,…,K}. In other words, ψ partitions the dimensions of $z^{t}$ into *K* subsets, where each subset is assigned to one of the *K* true factors.
  For a certain ψ, we can consider $p_{\phi }$ as the composition of two parts:
  - $p_{\phi ,shared}$: A shared part whose parameters affect all dimensions of ${\hat{z}}^{t+1}$;
  - $p_{\phi ,\psi_{k}},k\in 1\ldots K$: A set of *K* parts, where the parameters of the part with index *k* affect only the dimensions of ${\hat{z}}^{t+1}$ that are assigned to factor *k*.

We then say a model *M* has achieved Causal Mechanism Disentanglement with regard to distribution *D* if there exists an assignment function ψ such that two requirements hold: First, there exists a set of deterministic functions ${\left\{\eta_{k}:R^{\#\psi_{k}}\to R^{\zeta_{k}}\right\}}_{k=1}^{K}$ such that $\eta_{k}\left(z_{\psi_{k}}^{t}\right)=c_{k}^{t}$ for all k∈{1,…,K}, where $\psi_{k}=\left\{j|j\in \left\{1\ldots J\right\},\psi \left(j\right)=k\right\}$ corresponds to the dimensions of $z^{t}$ that are assigned to causal factor *k*, and $\#\psi_{k}$ is the number of elements in $\psi_{k}$. Second, the model’s transition prior $p_{\phi }$ accurately predicts the next-step latents:

(2) $$\begin{array}{c} \forall c^{t}:e_{\theta }\circ h\left(c_{1}^{t+1},\ldots ,c_{k}^{t+1}\right)=p_{\phi }\circ e_{\theta }\circ h\left(c_{1}^{t},\ldots ,c_{k}^{t}\right) \end{array}$$

Here, ∘ indicates the function composition: f∘g(x)=f(g(x)).

The first requirement corresponds to causal representation learning as defined in [11]. We use Causal *Factor* Disentanglement as a synonym for causal representation learning, as it concerns disentangling latent activations $z^{t}$ with regard to the true causal factors $c^{t}$. Furthermore, if $c_{j}^{t}\in Pa\left(c_{i}^{t+1}\right)$, $k\in \psi_{j}$ and $l\in \psi_{i}$, we simply write $z_{k}^{t}\in Pa\left(z_{l}^{t+1}\right)$.

In order to fulfill the first requirement (i.e., achieve Causal Factor Disentanglement), we use the CITRIS model [11]. To achieve Causal Mechanism Disentanglement with regard to the ID mechanisms, we fulfill the second requirement by training the model to accurately predict the next frame in the ID data.

#### 3.2.2. Relevance to Few-Shot Next-Frame Prediction

Informally, Causal Mechanism Disentanglement is about making distinct subsets of the parameters of $p_{\phi }$ responsible for modeling distinct true causal mechanisms $f_{t}$. If the Sparse Mechanism Shift assumption holds, successful Causal Mechanism Disentanglement entails that only the subset of parameters that modeled the shifted mechanisms needs to change in response. The parameters that model mechanisms that did not shift can remain the same. Causal Mechanism Disentanglement thus reduces the expected number of parameters that need to update.

Figure 1a illustrates this. It shows a target distribution that is shifted with regard to the one shown in Figure 1b that was caused by a single causal mechanism change, indicated in green. Gradient descent in a smaller parameter search space is then expected to adapt the model to a good solution more quickly, a property that has been empirically observed in [12].

#### 3.2.3. Shared Parameters

A restriction of our setup concerns shared parameters. Consider model parameters that are shared across all latent predictions (i.e., the shared parameters in the transition parameter $p_{\phi }$, as well as all parameters of the encoder $e_{\theta }$). If these shared parameters already ‘specialize’ to particular source-domain causal mechanisms, it restricts the extent to which the model can ‘get away’ with only updating the nonshared parameters. Addressing this specialization is an interesting avenue for future work.

## 4. Experimental Setup

In this section, we detail our benchmark datasets, the models we compare, and our evaluation metrics.

### 4.1. Datasets

#### 4.1.1. TRIS Datasets

Our benchmark is derived from the Temporal Intervened Sequences (TRIS) datasets [11]. We select these datasets for two reasons. First, they satisfy the property of being Mechanisms-Induced. They are generated as one long sequence of observable video frames, where the mechanisms dictate how the latent causal factors evolve from those at the previous timestep, and the rendering function determines how that translates into observed frames. Note that this entails that the underlying causal factors are typically not mutually independent.

The second reason for starting from the TRIS datasets is that they include extra information that allows a model to disentangle the underlying causal factors. This disentanglement is key to the way in which we exploit the Sparse Mechanism Shift assumption. Specifically, they include the presence of a binary soft-intervention value $\iota_{k}^{t+1}\in \left\{0,1\right\}$ for each factor *k* at each timestep t+1 that changes the natural mechanism $f_{k}$ into an intervened mechanism $f_{k}^{\iota }:Pa\left(c_{k}^{t+1}\right)\times u_{k}\to c_{k}^{t+1}$ if equal to 1. This kind of extra information can be realistic for learning agents, where the interventions might correspond to the actions of the agent (For example, the natural mechanism of a pie in a hot oven might be to transform a pie at one timestep into a slightly expanded pie at the next timestep, but if the agent intervenes by turning off the oven, the mechanism will take the same input pie but produce a smaller one over time).

In this work, we assume that for a given time *t*, the $\iota_{k}^{t}$ for different factors $c_{k}^{t}$ are sampled independently.

This extra information is also necessary to achieve Causal Factor Disentanglement.

The task for this dataset is then to predict the next-timestep frame $X^{t+1}$, based on the current-timestep frame $X^{t}$ and the intervention vector $\iota^{t+1}$.

**Figure 2:**
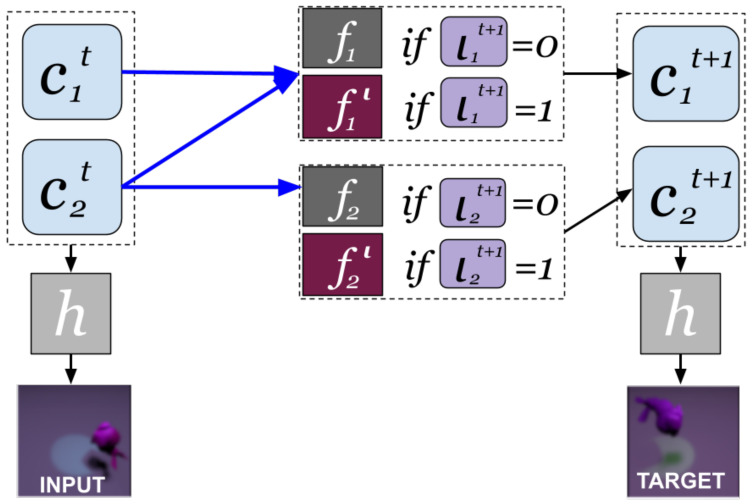
Temporal Intervened Sequence (TRIS) data setup for K=2 causal factors, for which $Pa\left(c_{1}^{t+1}\right)=\left\{c_{1}^{t},c_{2}^{t}\right\}$ and $Pa\left(c_{2}^{t+1}\right)=c_{2}^{t}$. The mechanism governing the evolution of the causal factors is the natural mechanism $f_{k}$ if $\iota_{k}^{t+1}=1$, and the intervened mechanism $f_{k}^{\iota }$ is otherwise.

Figure 2 shows an example for K=2, $Pa\left(c_{1}^{t+1}\right)=\left\{c_{1}^{t},c_{2}^{t}\right\}$ and $Pa\left(c_{2}^{t+1}\right)=\left\{c_{2}^{t}\right\}$.

**Figure A1:**
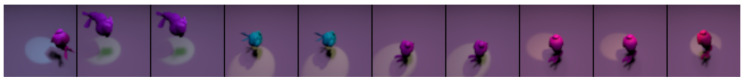
Example subsequence of the Shapes dataset.

**Figure A2:**
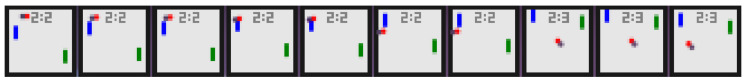
Example subsequence of the Pong dataset.

We use the two TRIS datasets reported in [11]: Temporal Causal3DIdent (Shapes) (For Shapes, we use the same object shapes as [11]: Cow [27], Head [28], Dragon [29], Hare [30], Armadillo [31], Horse [32], and Teapot [33]. We do not use the Teapot-only variant, but the variant with all seven shapes) and Pong. Both are synthetic datasets, where the true causal factors and mechanisms are known. Shapes consists of 250,000 frames at a resolution of 64 × 64. It displays a three-dimensional object varying in position/orientation/color, which makes it representative for real-life applications involving three-dimensional objects such as a robot manipulating certain household objects. Pong consists of 100,000 frames at a resolution of 32 × 32. It shows frames of an adaptation of the Atari game of Pong [34], making it relevant for applications that involve game-playing. We show examples of the Shapes and Pong datasets in Figure A1 and Figure A2 in Appendix A.

#### 4.1.2. SMS-TRIS Benchmark

We create out-of-distribution versions of these datasets by changing the causal mechanisms of one factor at a time. The ID mechanisms correspond to those in the original TRIS datasets. We generate nine OOD variants of the Shapes dataset and eight OOD variants of the Pong dataset, each of which differ from the original dataset in one mechanism. Following the original TRIS dataset, we generate each variant as one long sequence. For both Shapes and Pong, we generate 10,000 frames for each OOD variant.

For the Shapes dataset, the original causal mechanisms and the OOD-shifted mechanisms are shown in Table 1 for the position, rotation, and hue values.

For object hue, the mechanism can be described as trying to achieve a particular hue value depending on the object shape. The OOD-shifted-mechanism changes this target hue value, as shown in Table 2.

Table 3 shows the changes in the causal mechanisms of the factors in Pong.

In the SMS-TRIS benchmark, the latent causal factors correspond to human-interpretable factors such as color, position, etc. For disentanglement to benefit domain adaptation, human-interpretability is in itself not a required property of the factors that the model disentangles. Their only required property is that the distribution shift of interest is sparse in the space of mechanisms that generate them. An avenue for future work is to evaluate the effectiveness of disentanglement when latent factors are not human-interpretable.

### 4.2. Models

Our hypothesis is that we can improve the few-shot OOD prediction accuracy of a model by not only training it to predict accurately on the source-domain data, but also encouraging it to be disentangled. To achieve this disentanglement, we use the Causal-Factor-Disentanglement-encouraging extensions proposed in [11], which we refer to as ‘CITRIS CFD extensions’. We compare models with CITRIS CFD extensions (‘CITRIS’) to baseline models (‘Standard’) with the same backbone, but none of the extensions. As in [11], we do this for two backbone variations: an Auto-Encoder + Normalizing Flow (NF) and a Varatiational Auto-Encoder (VAE). This results in four models: CITRIS-NF, CITRIS-VAE, Standard-NF, and Standard-VAE.

Additionally, we compare the effect that disentanglement encouragement has with the effect of Deep Correlation Alignment (Deep CORAL [13]), an alternative method designed to improve the domain adaptation performance that is not designed to exploit the Sparse Mechanism Shift assumption.

We now detail the backbones, summarize the CITRIS CFD extensions, and explain the DeepCORAL baseline.

#### 4.2.1. Backbones

The Variational Auto-Encoder backbone consists of an encoder $e_{\theta }$, a decoder $d_{\iota }$, and a transition prior $p_{\phi }$. Following [11], the decoder $d_{\iota }$ is an approximation of the inverse encoder $e_{\theta }^{-1}$. Each of these components predict a probability distribution by predicting the mean and standard deviation of a normal distribution. We indicate the predicted probability distributions of these components as $P_{\theta }$, $P_{\iota }$, and $P_{\phi }$, respectively. The VAE objective then consists of a reconstruction term and a Kullback–Leibler (KL) divergence term:

(3) $$\begin{array}{c} \begin{array}{cc} L_{VAE}= & -E_{z^{t+1}∼P_{\theta }\left(z^{t+1}|X^{t+1}\right)}\left(\log P_{\iota }\left(X^{t+1}|z^{t+1}\right)\right)+\\ \; & E_{z^{t}∼P_{\theta }\left(z^{t}|X^{t}\right),z^{t+1}∼P_{\theta }\left(z^{t+1}|X^{t+1}\right)}\left(D_{KL}(P_{\theta }\left(z^{t+1}|X^{t+1}\right)\left|\right|P_{\phi }\left(z^{t+1}|z^{t},\iota^{t+1}\right)\right) \end{array} \end{array}$$

The Normalizing Flow backbone does not operate on $X^{t}$ directly. Rather, an autoencoder {$e_{AE},d_{AE}$} is first trained with a reconstruction objective (where $d_{AE}$ again approximates $e_{AE}^{-1}$), and the model then operates on the encoded images $y^{t}=e_{AE}\left(X^{t}\right)$. Another encoder $e_{\theta }$ is then trained to map $y^{t}$ to a $z^{t}$ that is intended to be disentangled with regard to the causal factors $c^{t}$. This latter encoder $e_{\theta }$ is implemented via an invertible multilayer normalizing flow (NF) network. The encoder $e_{\theta }$ is responsible for ensuring Causal Factor Disentanglement, i.e., the information about different true factors $c_{k}^{t}$ is stored in separate dimensions of $z^{t}$.

The transition prior part of the model ($p_{\phi }$) then predicts a mean and log standard deviation for the next timestep in the $z^{t}$-space and uses this to optimize for the Negative Log Likelihood (NLL) of $y^{t+1}$ after rescaling with the help of the Jacobian of the flow network $e_{\theta }$.

For both backbones, we follow [11] in using an autoregressive transition prior.

#### 4.2.2. CITRIS CFD Extensions

To achieve Causal Factor Disentanglement, CITRIS consists of a number of extensions to the basic KL-Divergence/NLL objective. We summarize the extensions here and refer to [11] for an in-depth explanation.

Three extensions are about restricting information about the input based on the learned assignment function ψ when predicting the NLL (for a Normalizing Flow) or KL-Divergence (for a VAE).

First, CITRIS restricts access to the information in the intervention vector $\iota^{t+1}$ during the prediction of a particular $z_{i}^{t+1}$. Specifically, it only allows access to the element in $\iota^{t+1}$ to which that particular $z_{i}^{t+1}$ is assigned by ψ. The motivation is that whatever properties of the observed data that the *encoder* stores in a particular $z_{i}^{t+1}$, the *transition prior* will need to be able to predict that property as accurately as possible using $z^{t}$ and *only one part of* $\iota^{t+1}$. Each part of $\iota^{t+1}$ contains information relevant for the prediction of properties corresponding to one true causal factor. Hence, the encoder allows the transition prior to make the best possible prediction of a dimension $z_{i}^{t+1}$ by letting that dimension contain only properties corresponding to the causal factor it is assigned to. This encourages the encoder to disentangle the information in its output $z^{t+1}$ with regard to the causal factors by making use of the information $\iota^{t+1}$ has on the causal factors.

Second, CITRIS restricts access to the information in the previous timestep $z^{t}$ during the prediction of a particular $z_{i}^{t+1}$. Consider the case where $z_{i}^{t+1}$ is assigned by ψ to the first element of $\iota^{t+1}$ and that first element in $\iota^{t+1}$ indicates whether the ’object-color’ was intervened on. If that first element of $\iota^{t+1}$ equals 1, the ’object-color’ was indeed intervened on, meaning that its value is no longer correlated with the previous frame. In that case, it is not useful for the model to let its predictions of the $z^{t+1}$-dimensions that are mapped by ψ to the ’object-color’ depend on the previous frame. Accordingly, CITRIS erases the information about the previous timestep for the prediction of a $z_{i}^{t+1}$ that is mapped by ψ to a causal factor for which $\iota^{t+1}$ equals 1.

Third, the transition prior is autoregressive *per group of $z^{t}$-dimensions assigned to the same factor*. Empirically, restricting the autoregression in this way is important for improving disentanglement.

Two further extensions add additional terms to the loss besides the KL-Divergence/NLL objective.

First is a target classifier: an added MLP trained to predict each element in the intervention vector $\iota^{t+1}$ from $z^{t}$ and different subsets of $z^{t+1}$. The encoder $e_{\theta }$ is then encouraged to allow the target classifier to be maximally accurate when predicting an element of $\iota^{t+1}$ from the subset of $z^{t+1}$ that matches it according to ψ, but less accurate (only up to predicting the average value) when predicting it from a nonmatching subset. Since this loss term is quite involved, we refer to appendix C.3.3 in [11] for its detailed description.

Second is a term that is intended for the situation when not all the information in the video frames is predicted by the causal factors that the model can disentangle with the help of the intervention vector. The term biases ψ to reserving some dimensions of $z^{t+1}$ for this ‘extra information’:

(4) $$L_{bias}=\lambda_{bias}\frac{1}{L}\sum_{l=1}^{L}\left(1-\frac{e^{\Psi_{0,l}}}{\sum_{j=0}^{K}e^{\Psi_{j,l}}}\right)$$

Here, $\lambda_{bias}$ is a hyperparameter (set to 0.01 in [11] and in this work), *L* is the number of dimensions of $z^{t+1}$, *K* is the number of causal factors, and Ψ is a K+1 by the *L* parameter matrix containing logits that are used to assign each of the *L* dimension of $z^{t+1}$ to a causal factor (rows 1 to K) or to reserve it for this ‘extra information’ (row 0).

The Standard models leave out all of the CITRIS CFD extensions. This means this model is no longer biased to be more disentangled than what is useful for the ID prediction objective.

The Standard model with the VAE backbone is equivalent to Identifiable VAE (iVAE [14]), in which the conditioning information consists of the previous timestep along with the intervention information.

#### 4.2.3. Deep CORAL

To place the impact of the CITRIS CFD extensions on few-shot domain adaptation into context, we compare its impact to that of an existing method designed for domain adaptation. For our problem setup, the method needs to be applicable to non-categorical prediction targets and needs to be able to work with access to only one source distribution. We select Deep CORAL [13] as an external baseline that is compatible with our problem setup.

Deep CORAL works by accessing the OOD data *during* ID training and encouraging the covariance matrices of latent activations of the ID and OOD batches to be close to each other. Since this is orthogonal to the CITRIS extensions, we report CITRISNF, Standard, CITRISNF + Deep CORAL, and Standard + Deep CORAL.

The variants using Deep CORAL cannot reuse the same ID-pretrained model for different OOD shifts. That is because they need access to the ID and OOD data *at the same time* during pretraining, so each pretrained model is specific to one OOD shift. Since we evaluate many OOD shifts, this significantly increases the computational requirements. Hence, we pretrain for a maximum of 50 epochs in the experiments that compare the effect of CFD encouragement to the effect of Deep CORAL, whereas we pretrain for 500 epochs in the other experiments. We select the pretrained checkpoint based on the best ID prediction performance.

### 4.3. Evaluation

To investigate the relation between Causal Factor Disentanglement (CFD), Causal Mechanism Disentanglement (CMD), and the OOD next-timestep-prediction performance, we report a number of metrics.

#### 4.3.1. Causal Factor Disentanglement (CFD)

To quantify the level of disentanglement of the latents ($z^{t}$) with regard to the true causal factors ($c^{t}$) for different models after ID training, we use the correlation metrics and triplet evaluation used in [11].

For the correlation metrics, a mapping $m_{k,j}$ is learned to predict each ${\hat{c}}_{k}^{t}=m_{k,j}\left(z_{\psi_{j}}^{t}\right)$ from each group of latents $z_{\psi_{j}}^{t},1\leq j\leq K$ (so not only from the ’matching’ group of latents $z_{\psi_{k}}^{t}$). Note that this mapping $m_{k,j}$ is not part of the model, but is learned purely for evaluation purposes. The correlation between the prediction $m_{k,j}\left(z_{\psi_{j}}^{t}\right)$ and the true factor $c_{k}^{t}$ is then reported. A well-disentangled model should have high correlation values for k=j, and low values otherwise. The $R^{2}$ coefficient of determination [35] and the Spearman’s rank correlation coefficient [36] are reported. Since the TRIS and SMS-TRIS datasets are generated sequentially, factors in each frame can be correlated, which can skew the interpretation of these correlation metrics. Hence, the correlation metrics are evaluated on independently sampled video frames. For the ‘Standard’ models, ψ is not learned. Hence, each latent dimension is assigned to the causal factor that it has the highest correlation with.

We also report the triplet distance, calculated as follows. A separate evaluation dataset is rendered consisting of triplets of frames: two randomly sampled images and a third image rendered using a mix of causal factors of the other two images. The model then computes the latents for the two original images, and a third latent is made by using the same mix of groups of latent dimensions assigned to a particular causal factor. The model is then tasked to decode this third latent to an image, which is compared to the rendered third image. The comparison is not reported in the pixel space but in the causal-factor space. To do so, a mapping from images $X^{t}$ to causal factors $c^{t}$ is learned in a supervised fashion. For categorical causal factors (such as shape), accuracy is used, while absolute difference is used for continuous variables. Each distance is normalized to fall between 0 and 1.

Note that both the independently sampled frames used for the correlation metrics and the triplets of frames used for the triplet distance do not consist of frames related over time through causal mechanisms. Hence, the same CFD score applies to both the in-domain and out-of-domain datasets.

Measuring Causal Factor Disentanglement is possible only because the true causal factors are known in the TRIS datasets. For real applications, we might not have access to the true causal factors. We are, however, still interested in measuring CFD to evaluate the effectiveness of encouraging CFD as a tool to reduce few-shot prediction error. Such a tool is useful for real applications, even if we cannot verify the level of disentanglement in real applications.

#### 4.3.2. Prediction Error

We report the OOD prediction error for different models after transfer learning. After pretraining the models on the ID data, we fine-tune them on the OOD data with a varying number of available OOD samples. We report the results for differing choices of the ID-training checkpoint to start fine-tuning from. We freeze $e_{\theta }$ during this fine-tuning and only train the transition prior: in line with the argument for Causal Mechanism Disentanglement made in Section 3, the model should be able to learn to predict the OOD data by adapting only (a subset of) the parameters of the transition prior. While the number of available samples varies, we fine-tune for a fixed number of epochs (50).

We evaluate the models on a held-out test set of the OOD data. We measure errors in the causal factor space, in the same way as for the triplet evaluation.

## 5. Results and Discussion

### 5.1. Causal Factor Disentanglement (CFD)

Table 4 shows the disentanglement metrics for both models for the Shapes and Pong datasets.

We are only able to reproduce the strong improvement in the level of disentanglement reported in [11] for the Shapes dataset and the NF backbone. Hence, we expect the CITRIS CFD extensions to improve few-shot accuracy only in that setting. For Shapes, the most challenging factors to disentangle were usually those concerning the rotation of the object. For Pong, the most challenging was to disentangle both paddle positions into separate dimensions, as their movements are very correlated.

### 5.2. Prediction Error

We show three perspectives on the prediction error. In the first perspective, we vary the pretraining epoch (between 1 and 500) used as a starting point for the few-shot domain adaptation and show the prediction error for one number of available shots (i.e., 200 shots). In the second perspective, we select one pretraining epoch (i.e., epoch 500) and show the prediction error for various numbers of available shots (i.e., 20, 100, 1000, and 10,000 shots). Finally, we also show a scatterplot comparing the CFD score to the prediction error at one number of shots (i.e., 100) and one pretraining epoch (i.e., 500).

#### 5.2.1. Varying Pretraining Epoch

**Figure 3:**
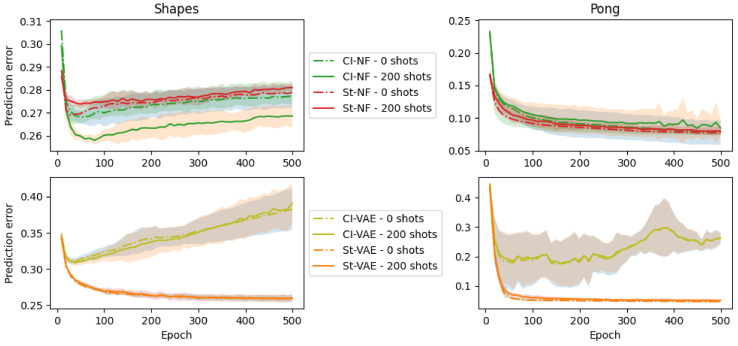
The 0-shot OOD error and 200-shot OOD error (lower is better) that a model achieves if the source-domain pretraining checkpoint at the epoch indicated on the x-axis is taken. The Standard models (St-VAE and St-NF) are shown in orange/red and the CITRIS models (CI-VAE and CI-NF) in green/olive. The left figures show results for the Shapes dataset and the right for the Pong dataset. The top figures show the Normalizing flow backbone and the bottom figures the VAE backbone. The line indicates the average of five runs, with the shaded areas indicating the standard deviation.

Figure 3 shows the evolution of the target-domain error with source-domain epochs for both SMS-TRIS datasets.

For Shapes and the NF backbone, we see that CITRIS-NF obtains the best performance. We also see that CITRIS-NF is the only variant for which the 200-shot performance (full lines) is significantly better than the 0-shot (dotted lines) performance. This is in line with our hypothesis that successful disentanglement improves few-shot domain adaptation.

For the VAE-models, the CITRIS-CFD extensions are not beneficial and Standard-VAE (which is equivalent to iVAE) is significantly better. This can be explained by the limited success of CITRIS-VAE in improving Causal Factor Disentanglement over Standard-VAE, as observed in Table 4.

For Pong, we also do not see that the CITRIS-CFD extensions are beneficial. This is again in line with the observation in Table 4 that the CITRIS-CFD extensions fail to improve Causal Factor Disentanglement. This indicates that finding more robust disentanglement encouragement is an avenue to more robustly improving few-shot domain adaptation prediction accuracy.

#### 5.2.2. Varying Number of Shots

**Figure 4:**
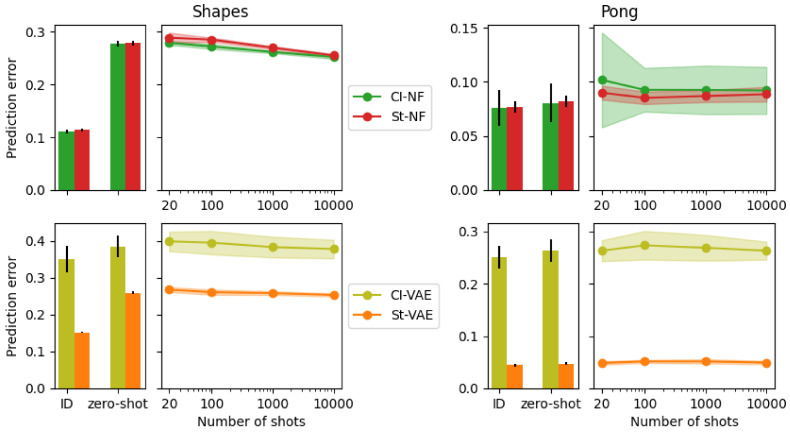
In-domain error, 0-shot error, and few-shot error of models after pretraining for 500 epochs. The Standard models are shown in red/orange and the CITRIS models in green/olive. The left figures show results for the Shapes dataset and the right for the Pong dataset. The top figures show the NF backbone and the bottom figures the VAE backbone. The average of five runs is shown, with the error bars/shaded areas indicating the standard deviation. The x-axis indicates in-domain or zero-shot out-of-domain for the bar charts and the number of shots used for the line plots.

Figure 4 also shows the error, but displays the number of shots on the x-axis, rather than the pretraining epoch as in Figure 3. The pretraining epoch is fixed to 500.

For Shapes, it confirms the observation from Figure 3 that CITRIS-NF sees the biggest improvement compared to its zero-shot performance. For Pong, we see that none of the models are able to improve over their zero-shot performance, indicating that adaptation in SMS-Pong is more challenging than in SMS-Shapes.

### 5.3. Causal Factor Disentanglement versus Prediction Error

**Figure 5:**
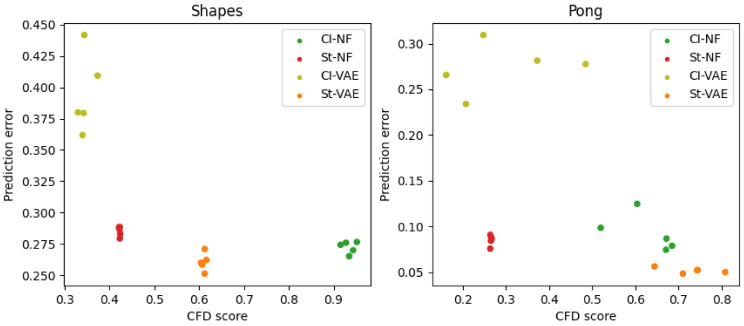
Correlation between few-shot prediction performance and Causal Factor Disentanglement. The y-axis shows 100-shot prediction error after pretraining for 500 epochs. The x-axis shows the CFD Score. Results for the Shapes dataset are on the left, and for Pong, on the right.

Figure 5 shows the correlation between few-shot prediction performance and Causal Factor Disentanglement.

It shows that while adding CITRIS does not consistently improve the CFD score, a higher CFD score is positively correlated with a lower prediction error, regardless of how it was achieved. This again indicates that finding more robust disentanglement encouragement is a promising avenue.

### 5.4. Comparison to Deep CORAL

**Figure 6:**
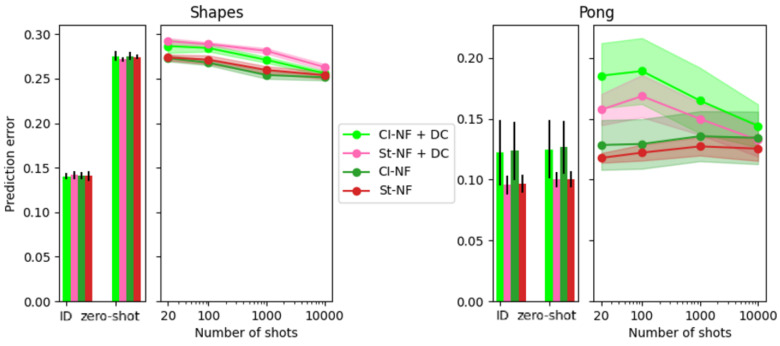
The effect of Deep CORAL on in-domain error, 0-shot error, and few-shot error of models after pretraining for 50 epochs. The Standard models are shown in shades of red and the CITRIS models in shades of green. The left figure shows results for the Shapes dataset and the right for the Pong dataset. The average of five runs is shown, with the error bars/shaded areas indicating the standard deviation. The x-axis indicates in-domain or zero-shot out-of-domain for the bar charts and the number of shots used for the line plots.

Figure 6 evaluates the effect of including the Deep CORAL objective. We only consider the Normalizing Flow backbone, as it showed the most consistent few-shot learning. Since using Deep CORAL is complementary to the use of CITRIS CFD encouragement, we show four variations: CITRIS-NF, CITRIS-NF + Deep CORAL, Standard-NF, and Standard-NF + Deep CORAL. The results show that the Deep CORAL objective is *not* helpful for the SMS-TRIS benchmark, highlighting the need for alternative methods to exploit the SMS assumption.

### 5.5. Discussion

We observe that CFD encouragement, using the CITRIS CFD extensions, does not improve the CFD score consistently across different backbones and datasets. This indicates that a more robust way of improving the CFD score is important when leveraging disentanglement for few-shot domain adaptation.

Our results indicate that CFD encouragement can help specifically in improving the relative improvement of *few-shot* performance over *zero-shot* performance, which is in line with the observations made in [12]. However, adding the CFD encouragement sometimes damages the zero-shot performance.

One factor that might be limiting the benefits of disentanglement is that shared parameters in the encoder and transition prior specialize to source-domain mechanisms. If that happens, the model cannot “get away” with only updating the nonshared parameters that are disentangled with regard to the shifted mechanism. Hence, it can be valuable to achieve disentanglement without being influenced by specific ID mechanisms.

## 6. Conclusions

This paper proposed a method to improve accuracy in the challenging problem of transfer learning for few-shot video prediction. Specifically, we aim to exploit the situation where the source and target domains are related through a Sparse Mechanism Shift. In this situation, we study the idea of encouraging the model to be disentangled with regard to causal mechanisms, allowing it to improve their few-shot prediction performance. We argue that successful Causal Representation Learning can enable such disentanglement. To experimentally investigate this connection, we created a novel benchmark called SMS-TRIS that consists of datasets with source-domain and target-domain variants that vary by a single distribution shift. Using this benchmark, we compare the effect of Causal Representation Learning using the CITRIS extensions proposed in [11] on a number of baselines.

Our results suggest that including CITRIS extensions benefits few-shot target-domain accuracy *if* those extensions allow the model to reach a sufficient level of Causal Factor Disentanglement on the source-domain data. Our experiments also indicate that few-shot learning in general is a hard problem and that it is often hard to beat the simple baseline of not using the few available target-domain samples at all.

We hope this work can spark research that further looks into the best ways to leverage the Sparse Mechanism Shift assumption for few-shot domain adaptation.

## Figures and Tables

**Table 1:** OOD mechanisms for position, rotation, and hue values. They cover different kinds of distribution shifts: ones that keep the same input, ones that depend on different inputs, and one in which a relation is changed from linear to non-linear. Red text highlights the difference between ID mechanism and OOD mechanism. The function $f\left(a,b,c\right)=\frac{a-b}{2}+c$ and *u*-variables are independently sampled from a Gaussian distribution as described in [11]. For HUE_O__ID_GOAL and HUE_O__OOD_GOAL, see ID object hue goal and OOD object hue goal, respectively, in Table 2.

Factor	ID Mechanism	OOD Mechanism
$pos\_x^{t+1}$	$f(1.5\cdot \sin\left(rot\_\beta^{t}\right),pos\_x^{t},u_{X}^{t})$	$f(1.5\cdot \cos\left(rot\_\beta^{t}\right),pos\_x^{t},u_{X}^{t})$
$pos\_y^{t+1}$	$f(1.5\cdot \sin\left(rot\_\alpha^{t}\right),pos\_y^{t},u_{y}^{t})$	$f(-1.5\cdot \cos\left(rot\_\alpha^{t}\right),pos\_y^{t},u_{y}^{t})$
$pos\_z^{t+1}$	$f(1.5\cdot \cos\left(rot\_\alpha^{t}\right),pos\_z^{t},u_{z}^{t})$	$f(1.5\cdot \sin\left(rot\_\beta^{t}\right),pos\_z^{t},u_{z}^{t})$
$rot\_\alpha^{t+1}$	$f(hue\_b,rot\_\alpha^{t},u_{\alpha }^{t})$	$f(hue\_o,rot\_\alpha^{t},u_{\alpha }^{t})$
$rot\_\beta^{t+1}$	$f(hue\_o,rot\_\beta^{t},u_{\beta }^{t})$	$f(hue\_b,rot\_\beta^{t},u_{\beta }^{t})$
$rot\_s^{t+1}$	$f(atan2\left(pos\_x^{t+1},pos\_y^{t+1}\right),rot\_s^{t},u_{rs}^{t})$	$f(atan2\left(pos\_y^{t+1},pos\_z^{t+1}\right),rot\_s^{t},u_{rs}^{t})$
$hue\_s^{t+1}$	$f(2\pi -hue\_b^{t},hue\_s^{t},u_{hs}^{t})$	$f(2\pi -hue\_b^{t}\cdot {\mathrm{hue\_s}}^{t},hue\_s^{t},u_{hs}^{t})$
$hue\_b^{t+1}$	$hue\_b^{t}+u_{hb}^{t}$	$hue\_b^{t}+u_{hb}^{t}+{\mathrm{hue\_o}}^{t}$
$hue\_o^{t+1}$	$f(\mathrm{HUE}O\_\mathrm{ID}\_\mathrm{GOAL},hue\_o^{t},u_{ho}^{t})$	$f(\mathrm{HUE}O\_\mathrm{OOD}\_\mathrm{GOAL},hue\_o^{t},u_{ho}^{t})$

**Table 2:** OOD mechanisms for object hue. We choose the changes so that the inherent difficulty is not changed.

Object Shape	ID Object Hue Goal	OOD Object Hue Goal
Teapot	0	π
Armadillo	$\frac{1}{5}\cdot 2\pi$	0
Hare	avg(hue spot, hue back)	avg(hue spot, hue back) + π
Cow	$\frac{2}{5}\cdot 2\pi$	$\frac{3}{5}\cdot 2\pi$
Dragon	avg(hue spot, hue back) + π	avg(hue spot, hue back)
Head	$\frac{3}{5}\cdot 2\pi$	$\frac{2}{5}\cdot 2\pi$
Horse	$\frac{4}{5}\cdot 2\pi$	$\frac{4.5}{5}\cdot 2\pi$

**Table 3:** Changes in the causal mechanisms of the factors in Pong. Since a Pong timestep plays out in multiple stages, we need to ensure that a mechanism change always only affects only one variable given its causal parents.

Factor	OOD Mechanism Change
Ball x-position	The ball now teleports horizontally over a middle section whose width is half the inter-paddle horizontal distance.
Ball y-position	The ball now teleports vertically over a middle section whose width is one-fifth of the inter-wall vertical distance.
Ball velocity direction	When a ball collides with a paddle, instead of only having the x-component of its velocity direction flipped, now the y-component also flips, flipping the velocity direction by 180 degrees.
Ball velocity magnitude	Ball velocity is doubled when in the lower half of the playing field.
Paddle left y-position	After a paddle–ball collision, the paddle now teleports a distance equal to half the inter-wall vertical distance up, if the collision was in the lower half, and down, if the collision was in the upper half.
Paddle right y-position	Same as for paddle left y-position.
Score left	When a score of 5 is reached, the score now resets to 1 instead of 0.
Score right	Same as for score left.

**Table 4:** Disentanglement metrics for the Shapes and Pong data. $R^{2}$ is the $R^{2}$ coefficient of determination and Sp is Spearman’s rank correlation coefficient. *diag* corresponds to the average correlation score of the predicted causal factor $m_{k,k}\left(z_{\psi_{k}}^{t}\right)$ and its matching true factor $c_{k}^{t}$ (best value is 1); *offdiag* corresponds to the average of the maximum correlation score of a predicted causal factor $m_{i,k}\left(z_{\psi_{k}}^{t}\right)$ and a non-matching true factor $c_{i}^{t}$, i≠k (best value is 0). For triplet distances, the mean over all causal factors is reported (Triplet mean). CFD score is the average of all other metrics (using 1 minus [metric] for the metrics for which lower is better).

	Model	$R^{2}$ Diag ↑	$R^{2}$ Sep ↓	Sp Diag ↑	Sp Sep ↓	Triplet Mean ↓	CFD Score ↑
**Shapes**	CITRIS-NF	0.95 ± 0.011	0.08 ± 0.020	0.95 ± 0.017	0.10 ± 0.016	0.05 ± 0.008	0.93 ± 0.014
	Standard-NF	0.26 ± 0.001	0.62 ± 0.003	0.29 ± 0.001	0.61 ± 0.004	0.21 ± 0.000	0.42 ± 0.001
	CITRIS-VAE	0.63 ± 0.022	0.25 ± 0.062	0.63 ± 0.028	0.28 ± 0.050	0.34 ± 0.010	0.68 ± 0.031
	Standard-VAE	0.60 ± 0.016	0.46 ± 0.015	0.61 ± 0.014	0.47 ± 0.004	0.23 ± 0.001	0.61 ± 0.005
**Pong**	CITRIS-NF	0.64 ± 0.064	0.39 ± 0.046	0.62 ± 0.068	0.41 ± 0.045	0.31 ± 0.168	0.63 ± 0.070
	Standard-NF	0.13 ± 0.004	0.85 ± 0.006	0.13 ± 0.003	0.85 ± 0.002	0.25 ± 0.001	0.26 ± 0.001
	CITRIS-VAE	0.77 ± 0.085	0.25 ± 0.118	0.77 ± 0.086	0.31 ± 0.136	0.26 ± 0.030	0.75 ± 0.081
	Standard-VAE	0.83 ± 0.124	0.37 ± 0.081	0.83 ± 0.115	0.39 ± 0.054	0.25 ± 0.001	0.73 ± 0.060

## Data Availability

Data are contained within the article.

## Appendix A. Example Subsequence of Shapes and Pong Datasets

Figure A1 and Figure A2 show example subsequences of the Shapes and Pong datasets, respectively.

## References

[1] Filos A., Tigkas P., McAllister R., Rhinehart N., Levine S., Gal Y. Can autonomous vehicles identify, recover from, and adapt to distribution shifts?. Proceedings of the International Conference on Machine Learning, PMLR.

[2] Guariso G., Nunnari G., Sangiorgio M. (2020). Multi-step solar irradiance forecasting and domain adaptation of deep neural networks. Energies.

[3] Rothfuss J., Ferreira F., Aksoy E.E., Zhou Y., Asfour T. (2018). Deep Episodic Memory: Encoding, Recalling, and Predicting Episodic Experiences for Robot Action Execution. IEEE Robot. Autom. Lett..

[4] Teshima T., Sato I., Sugiyama M. Few-shot Domain Adaptation by Causal Mechanism Transfer. Proceedings of the 37th International Conference on Machine Learning, PMLR.

[5] Arjovsky M., Bottou L., Gulrajani I., Lopez-Paz D. (2019). Invariant Risk Minimization. arXiv.

[6] Liu C., Sun X., Wang J., Tang H., Li T., Qin T., Chen W., Liu T.Y. (2021). Learning causal semantic representation for out-of-distribution prediction. Adv. Neural Inf. Process. Syst..

[7] Wang R., Yi M., Chen Z., Zhu S. Out-of-distribution Generalization with Causal Invariant Transformations. Proceedings of the IEEE/CVF Conference on Computer Vision and Pattern Recognition, CVPR 2022.

[8] Schölkopf B., Locatello F., Bauer S., Ke N.R., Kalchbrenner N., Goyal A., Bengio Y. (2021). Toward causal representation learning. Proc. IEEE.

[9] Kozhubaev Y., Ovchinnikova E., Viacheslav I., Krotova S. (2023). Incremental Machine Learning for Soft Pneumatic Actuators with Symmetrical Chambers. Symmetry.

[10] Lopez R., Tagasovska N., Ra S., Cho K., Pritchard J., Regev A. Learning Causal Representations of Single Cells via Sparse Mechanism Shift Modeling. Proceedings of the Second Conference on Causal Learning and Reasoning, PMLR.

[11] Lippe P., Magliacane S., Löwe S., Asano Y.M., Cohen T., Gavves S. Citris: Causal identifiability from temporal intervened sequences. Proceedings of the International Conference on Machine Learning, PMLR.

[12] Bengio Y., Deleu T., Rahaman N., Ke N.R., Lachapelle S., Bilaniuk O., Goyal A., Pal C.J. A Meta-Transfer Objective for Learning to Disentangle Causal Mechanisms. Proceedings of the 8th International Conference on Learning Representations, ICLR 2020.

[13] Sun B., Saenko K. Deep CORAL: Correlation Alignment for Deep Domain Adaptation. Proceedings of the Computer Vision—ECCV 2016 Workshops.

[14] Khemakhem I., Kingma D.P., Monti R.P., Hyvärinen A., Chiappa S., Calandra R. Variational Autoencoders and Nonlinear ICA: A Unifying Framework. Proceedings of the 23rd International Conference on Artificial Intelligence and Statistics.

[15] Kim H., Mnih A., Dy J.G., Krause A. Disentangling by Factorising. Proceedings of the 35th International Conference on Machine Learning, ICML 2018.

[16] Lu C., Wu Y., Hernández-Lobato J.M., Schölkopf B. (2021). Nonlinear Invariant Risk Minimization: A Causal Approach. arXiv.

[17] Krueger D., Caballero E., Jacobsen J.H., Zhang A., Binas J., Zhang D., Priol R.L., Courville A. Out-of-Distribution Generalization via Risk Extrapolation (REx). Proceedings of the 38th International Conference on Machine Learning. PMLR.

[18] Rojas-Carulla M., Scholkopf B., Turner R., Peters J. (2018). Invariant Models for Causal Transfer Learning. J. Mach. Learn. Res..

[19] Shu R., Bui H.H., Narui H., Ermon S. A DIRT-T Approach to Unsupervised Domain Adaptation. Proceedings of the 6th International Conference on Learning Representations, ICLR 2018.

[20] Yoon J., Kang D., Cho M. Semi-supervised domain adaptation via sample-to-sample self-distillation. Proceedings of the IEEE/CVF Winter Conference on Applications of Computer Vision.

[21] Peng K., Wen D., Schneider D., Zhang J., Yang K., Sarfraz M.S., Stiefelhagen R., Roitberg A. (2023). FeatFSDA: Towards Few-shot Domain Adaptation for Video-based Activity Recognition. arXiv.

[22] Xu Y., Yang J., Zhou Y., Chen Z., Wu M., Li X. (2023). Augmenting and Aligning Snippets for Few-Shot Video Domain Adaptation. arXiv.

[23] Jiang J., Ji Y., Wang X., Liu Y., Wang J., Long M. Regressive domain adaptation for unsupervised keypoint detection. Proceedings of the IEEE/CVF Conference on Computer Vision and Pattern Recognition.

[24] Lang C., Cheng G., Tu B., Li C., Han J. (2023). Base and meta: A new perspective on few-shot segmentation. IEEE Trans. Pattern Anal. Mach. Intell..

[25] Tian Z., Zhao H., Shu M., Yang Z., Li R., Jia J. (2020). Prior guided feature enrichment network for few-shot segmentation. IEEE Trans. Pattern Anal. Mach. Intell..

[26] Liu H., Tam D., Muqeeth M., Mohta J., Huang T., Bansal M., Raffel C. Few-Shot Parameter-Efficient Fine-Tuning is Better and Cheaper than In-Context Learning. Proceedings of the NeurIPS.

[27] Crane K. Keenan’s 3D Model Repository. https://www.cs.cmu.edu/~kmcrane/Projects/ModelRepository/.

[28] Rusinkiewicz S., DeCarlo D., Finkelstein A., Santella A. Suggestive Contour Gallery. https://gfx.cs.princeton.edu/proj/sugcon/models/.

[29] Curless B., Levoy M. A volumetric method for building complex models from range images. Proceedings of the 23rd Annual Conference on Computer Graphics and Interactive Techniques.

[30] Turk G., Levoy M. Zippered polygon meshes from range images. Proceedings of the 21st Annual Conference on Computer Graphics and Interactive Techniques.

[31] Krishnamurthy V., Levoy M. Fitting smooth surfaces to dense polygon meshes. Proceedings of the 23rd Annual Conference on Computer Graphics and Interactive Techniques.

[32] Praun E., Finkelstein A., Hoppe H. Lapped textures. Proceedings of the 27th Annual Conference on Computer Graphics and Interactive Techniques.

[33] Newell M.E. (1975). The Utilization of Procedure Models in Digital Image Synthesis. Ph.D. Thesis.

[34] Bellemare M.G., Naddaf Y., Veness J., Bowling M. (2013). The arcade learning environment: An evaluation platform for general agents. J. Artif. Intell. Res..

[35] Wright S. (1921). Correlation and causation. J. Agric. Res..

[36] Spearman C. (1987). The proof and measurement of association between two things. Am. J. Psychol..

